# Social Media and Language Processing: How Facebook and Twitter Provide the Best Frequency Estimates for Studying Word Recognition

**DOI:** 10.1111/cogs.12392

**Published:** 2016-08-01

**Authors:** Amaç Herdağdelen, Marco Marelli

**Affiliations:** ^1^ Facebook; ^2^ Center for Mind‐Brain ScienceUniversity of Trento

**Keywords:** Frequency effects, Social media, Lexical decision, Text corpora

## Abstract

Corpus‐based word frequencies are one of the most important predictors in language processing tasks. Frequencies based on conversational corpora (such as movie subtitles) are shown to better capture the variance in lexical decision tasks compared to traditional corpora. In this study, we show that frequencies computed from social media are currently the best frequency‐based estimators of lexical decision reaction times (up to 3.6% increase in explained variance). The results are robust (observed for Twitter‐ and Facebook‐based frequencies on American English and British English datasets) and are still substantial when we control for corpus size.

## 1 Introduction

Word frequency is arguably the most important determinant of reaction times in word‐recognition tasks (Howes & Solomon, 1951). Thus, obtaining reliable word frequency estimates is an important endeavor in cognitive sciences and psycholinguistics (Brysbaert & New, 2009). Substantial word frequency effects have been observed in a variety of tasks ranging from naming latencies (Carroll & White, 1973), to fixation durations in eye‐tracking experiments (Juhasz & Rayner, 2006), to blood oxygenation level in neuroimaging studies (Chee, Venkatraman, Westphal, & Siong, 2003). However, the most frequently used task in which word frequency effects are crucial is the lexical decision, in which participants are presented with letter strings and asked to judge, as fast as possible, whether the string represents a real word or not. In these conditions, typically, reaction times of participants are shorter in recognizing high‐frequency words as opposed to low‐frequency words. Although the interpretation of the word frequency effect is still debated (Baayen, 2010), the reliability of the empirical effect is beyond doubt. Given the pervasiveness of word frequency effects, it is crucial to control for word frequency in cognitive science and neuroscience experiments involving language.

Although alternative methods have been used (e.g., subjective frequency ratings, Balota, Pilotti, & Cortese, 2001), the most widespread approach in collecting word frequencies is to process large text collections (*corpora*) and count word occurrences within them. However, corpus‐based approaches introduce a new question: Which corpus should one use? The source corpus obviously has an impact on the quality of the obtained frequency measures, where “quality” is defined here as the ability to capture data variance that is cognitively relevant. That is, if word frequency has to explain language processing, the source corpus should preferably represent the language experience of speakers.

Historically, the most popular word frequency datasets used in psycholinguistics, such as the Kucera and Francis norms (Kučera & Francis, 1967) and CELEX (Baayen, Piepenbrock, & Gulikers, 1995), were based on traditional corpora. Traditional corpora mostly include written samples of newspapers, textbooks, novels, and magazines. These provide a very limited approximation of speakers' experience. This type of text is usually heavily edited, uses exaggerated lexical variation, and is focused on few, limited topics detached from everyday social interactions (Brysbaert & New, 2009). In other words, they are characterized by a register that is very different from the spontaneous, natural usage of language.

In this perspective, the World Wide Web can be exploited as potential source of linguistic data that are closer to everyday experience. Burgess and Livesay (1998) proposed to employ user groups as supplies of frequency data. These groups collect unsupervised discussions between different users about a number of topics and can thus provide examples of linguistics interactions whose register is closer to natural language usage. Indeed, these frequency data (released by Balota et al., 2007) proved to be a reliable predictor of lexical decision latencies (Burgess & Livesay, 1998; Brysbaert & New, 2009). More recently, a number of frequency databases have been developed based on television subtitles. This approach has been extremely successful in predicting response times, and now subtitle frequency databases are available for a number of different languages (Brysbaert & New, 2009; Cai & Brysbaert, 2010; Dimitropoulou, Duñabeitia, Avilés, Corral, & Carreiras, 2010; Keuleers, Brysbaert, & New, 2010; Brysbaert et al., 2011; Vega, Nosti, Gutiérrez, & Brysbaert, 2011; Mandera, Keuleers, Wodniecka, & Brysbaert, 2014; van Heuven, Mandera, Keuleers, & Brysbaert, 2014). One explanation why subtitle databases are better predictors of word processing is that they are closer to natural language use than traditional corpora, providing example of dialogues during social interactions.

In this study, we take the tapping‐more‐natural‐context approach one step further and test Facebook and Twitter as sources for frequency norms to be used in psycholinguistic experiments. These social media present advantages over both newsgroups and subtitles. Compared to the newsgroups, Facebook and Twitter provide examples of language productions that are not restricted by specific topics. Compared to subtitles, they reflect spontaneous productions of normal language speakers, rather than the scripted and edited material that mostly constitutes subtitle databases. Moreover, thanks to their popularity, social media provide an always increasing quantity of linguistic data in many different languages, obtained through a very large sample of speakers. Although they are being extensively studied in computational linguistics (Eisenstein, O'Connor, Smith, & Xing, 2014; Rosenthal, Nakov, Ritter, & Stoyanov, 2014; Xu, Callison‐Burch, & Dolan, 2015), they remain an underused resource for psycholinguistic purposes (but see Gimenes & New, in press).

The present paper describes the first extensive investigation in these regards, demonstrating that word frequencies based on Facebook and Twitter data significantly outperform previously suggested word frequency norms in explaining lexical decision reaction times for both British and American English. In particular, even against a very strong baseline model including the state‐of‐the‐art frequency norms and word‐form properties (number of syllables and letters in the word), adding social media frequencies to the model increases the explained variance in reaction times by 3.7% points for British English and 1.5% points for American English. We also provide evidence that the superior predictive power of social media frequencies is not just due to increased corpora size (although larger corpora results in better performance), but to an overall better alignment across different linguistic categories of words.

## 2 Corpora

The present section briefly describes the source corpora at the basis of the frequency norms we will consider in the empirical testing.

### 2.1 Rovereto Twitter Corpus

The Rovereto Twitter Corpus (RTC, http://clic.cimec.unitn.it/amac/twitter_ngram/) is an n‐gram frequency corpus of tweets collected between December 2010 and July 2011 (Herdağdelen, 2013). The corpus is based on 75 million English public tweets that were obtained from Twitter, using the publicly available feed. RTC employed a cutoff frequency of three to remove rare lexical occurrences, leaving us with 1.17 billion tokens. We consider the token (unigram) frequencies and the number of distinct users who mentioned the word in the sample (user count).

### 2.2 Facebook word frequencies

In order to construct the Facebook word frequency norms, we sampled a random collection of anonymized, publicly available English posts that were created between November 2014 and January 2015. By separating the content created in the United States and Great Britain, we obtained two locale‐specific corpora. Each corpus consisted of approximately 1 billion tokens (1.10 billion for the American corpus and 1.18 billion for the British corpus).

The text was aggregated and tokenized automatically. No individually identifiable information was visible to researchers. After tokenization, number of occurrences and number of unique users who mentioned the words were computed. Henceforth, regular word frequency values will be referred to as FB‐US for the American corpus and FB‐UK for the British corpus. User count values will be denoted by the “UC” suffix.

In order to evaluate the impact of corpus size on the validity of the frequency norms, we also created down‐sampled versions of both RTC and FB corpora with sizes ranging from roughly 5 million to roughly 500 million tokens.

### 2.3 Subtitle‐based corpora

Subtitle‐based word frequencies provide the state‐of‐the‐art results in predicting lexical decision reaction times. In this study, we focused on two subtitle‐based datasets, SUBTLEX‐UK (http://crr.ugent.be/archives/1423, van Heuven et al., 2014) and SUBTLEX‐US (http://www.ugent.be/pp/experimentele-psychologie/en/research/documents/subtlexus, Brysbaert & New, 2009), collecting frequency norms for British English and American English, respectively.

SUBTLEX‐UK is a word frequency dataset based on the subtitles of 45,099 BBC broadcasts. It contains 201.3 million tokens. SUBTLEX‐US is similarly based on subtitles from US television series and films and contains 51 million tokens. Each database includes norms for word frequency and contextual diversity (CD), the latter defined as the number of unique programs that contain a given word.

### 2.4 Other frequency norms

HAL frequency norms (Burgess & Livesay, 1998; made available by Balota et al., 2007) were gathered across 3,000 Usenet newsgroups during February 1995, mostly in American English. Information concerning corpus size is quite inconsistent across different documents; the most recent report estimates about 400 million tokens (http://elexicon.wustl.edu).

CELEX (Baayen et al., 1995) is widely used in the word‐recognition literature. Its frequency norms are based on a corpus of 17.9 million tokens, based on samples of both written and spoken British English.

The British National Corpus (www.natcorp.ox.ac.uk) is a 100‐million‐word collection of examples of written and spoken language. Documents are sampled from a wide range of sources, designed to provide a faithful representation of British English in the late 20th century.

## 3 Procedures

In a series of analyses, we compare RTC‐ and FB‐based frequency norms to previously published norms. Following the established practice in the field, the performance of each norm was assessed using (a) Pearson correlations between frequencies and reaction times (RTs) and (b) the variance explained (in terms of *R* ^2^) by a linear model between the logarithm of frequencies and logarithm of RTs (e.g., log(RT)~log(frequency + 1)). Number of characters and syllables in the word were included as linear covariates in these models.

Reaction times were extracted from megastudies, a research approach increasingly popular in psycholinguistics (Keuleers & Balota, 2015). Megastudies focus on collecting behavioral responses on a large number of lexical items, without a specific scientific question guiding the endeavor. The resulting datasets can then be used for the large‐scale testing of scientific hypotheses and resources. Examples of applications of megastudies include investigation of individual differences in language processing (Yap, Balota, Sibley, & Ratcliff, 2012), validation of newly proposed measures (Yarkoni, Balota, & Yap, 2008; Marelli, Amenta, & Crepaldi, 2015), parameter setting in modeling (Shaoul & Westbury, 2010), and evaluation of computational systems (Baayen, Milin, Đurđević, Hendrix, & Marelli, 2011; Marelli & Baroni, 2015). Megastudies have also become the instrument of choice for the evaluation of frequency norms in a psycholinguistic perspective, providing the opportunity to test norm performance on a large number of words. Most megastudies are based on the lexical decision paradigm, where the participants are asked to decide whether a written letter string is an existing word by pressing buttons on a response box. Response latencies are automatically collected, and averaged across participants. For this study, we relied on visual lexical decision latencies included in the English Lexicon Project (ELP, Balota et al., 2007) and the British Lexicon Project (BLP, Keuleers, Lacey, Rastle, & Brysbaert, 2012). ELP includes response‐times and word‐naming latencies for 40,481 words, collected through the testing of 816 American‐English speakers. BLP includes response‐times' latencies for 28,730 words, collected through the testing of 78 British‐English speakers.

Following Brysbaert & New (2009), we selected as test items monosyllabic and disyllabic words that were correctly recognized by at least 66% of the participants in the megastudy. BLP contains only monosyllabic and disyllabic words (although we identified and removed 56 words with more than two syllables in the dataset). Concerning the ELP data, we filtered out all words with more than two syllables (in the Appendix we also report results on the complete ELP dataset). Since ELP contains words with mixed cased letters, we also filtered out all words with a capital cased letter in the corresponding set. As a result, we obtained two test sets. The former, based on ELP, included 17,280 words and was used to evaluate the performance of HAL, RTC, SUBTLEX‐US, and FB‐US norms. The latter, based on BLP, included 20,458 words and was used to evaluate the performance of CELEX, BNC, RTC, SUBTLEX‐UK, and FB‐UK norms.

Tables 1 and 2 report correlation matrices including frequency norms from the considered corpora along with response lantencies from ELP and BLP.

**Table 1:** Spearman correlations between frequency predictors and reaction times for American English

	RT	RTC	FB‐US	HAL	SUBTLEX‐US
RTC	−0.694				
FB‐US	−0.693	0.950			
HAL	−0.663	0.861	0.851		
SUBTLEX‐US	−0.672	0.891	0.891	0.851	
SUBTLEX‐US (CD)	−0.679	0.900	0.903	0.853	0.991

**Table 2:** Spearman correlations between frequency predictors and reaction times for British English

	RT	RTC	FB‐UK	BNC	CELEX	SUBTLEX‐UK
RTC	−0.714					
FB‐UK	−0.709	0.922				
BNC	−0.664	0.771	0.793			
CELEX	−0.650	0.747	0.763	0.936		
SUBTLEX‐UK	−0.694	0.858	0.887	0.888	0.845	
SUBTLEX‐UK (CD)	−0.701	0.865	0.892	0.901	0.863	0.992

## 4 Results

### 4.1 Predictive power

Social media frequencies explain more variance in lexical decision RTs than the state‐of‐the‐art frequency norms and have a higher correlation with the reaction times.

The results of the overall comparison are reported in Table 3 (BLP set) and in Table 4 (ELP set). Concerning RTC norms, their correlations with BLP RTs are significantly higher than those of CELEX (*z* = 10.51, *p* = .0001), BNC (*z* = 8.65, *p* = .0001), SUBTLEX‐UK (*z* = 3.73, *p* = .0002), and SUBTLEX‐UK CD (*z* = 2.07, *p* = .0385); their correlations with ELP RTs are significantly higher than those of HAL (*z* = 4.61, *p* = .0001), SUBTLEX‐US (*z* = 5.25, *p* = .0001), and SUBTLEX‐US CD (*z* = 3, *p* = .0027). Concerning FB‐UK norms, their correlations with BLP RTs are significantly higher than those of CELEX (*z* = 10.51, *p* = .0001), BNC (*z* = 8.65, *p* = .0001), SUBTLEX‐UK (*z* = 3.73, *p* = .0002), and SUBTLEX‐UK CD (*z* = 2.07, *p* = .0385). Concerning FB‐US norms, their correlations with ELP RTs are significantly higher than those of HAL (*z* = 4.78, *p* = .0001), SUBTLEX‐US (*z* = 5.42, *p* = .0001), and SUBTLEX‐US CD (*z* = 3.17, *p *= .0015). The significance of the difference between each pair of correlation coefficients was assessed using the Fisher *r*‐to‐*z* transformation.

**Table 3:** Absolute correlations and explained variance of various measures with respect to the response latencies from the British Lexicon Project

Corpus	Pearson Correlation	*R* ^2^
CELEX	0.627	0.409
BNC	0.638	0.422
SUBTLEX‐UK	0.666	0.459
SUBTLEX‐UK CD	0.675	0.471
FB‐UK	0.684	0.483
FB‐UK UC	0.686	0.486
RTC	0.686	0.487
RTC UC	0.686	0.487
Baseline		0.489
Baseline + FB‐UK		0.515
Baseline + RTC		0.522
Baseline + FB‐UK + RTC		0.525

Notes

CD, contextual diversity; UC, user count. Frequency values and response latencies are log‐transformed.

**Table 4:** Absolute correlations and explained variance of various measures with respect to the response latencies from the English Lexicon Project

Corpus	Pearson Correlation	*R* ^2^
HAL	0.646	0.429
SUBTLEX‐US	0.642	0.430
SUBTLEX‐US CD	0.656	0.448
RTC	0.673	0.467
RTC UC	0.674	0.467
FB‐US	0.674	0.468
FB‐US UC	0.675	0.469
Baseline		0.495
Baseline + RTC		0.506
Baseline + FB‐US		0.507
Baseline + FB‐US + RTC		0.509

Notes

CD, contextual diversity; UC, user count. Frequency values and response latencies are log‐transformed.

In the BLP dataset (Table 3), FB‐UK and RTC explain 1.5% and 1.6% more variance than SUBTLEX‐UK CD. In the ELP dataset (Table 4), FB‐US and RTC explains 2.0% and 1.9% more variance than SUBTLEX‐US CD. We computed competitive baseline models which incorporate previously reported frequency norms, along with formal properties of the word (as additive effects in the regression analysis). For the BLP set, the baseline includes, in addition to the number of characters and syllables, BNC and CELEX frequencies, SUBTLEX‐UK frequencies, and SUBTLEX‐UK CD values. Similarly, the baseline for ELP includes number of characters and syllables, HAL and SUBTLEX‐US frequencies, and SUBTLEX‐US CD values. In BLP, the baseline model explains 48.9% of the variance. When we introduce RTC and FB‐UK, the variance explained increases to 52.5%, with a significant improvement of 3.6% point (χ^2^(2) = 7.92, *p* = .0001). In ELP, the baseline explains 49.51% of the response‐time variance. When we introduce FB‐US and RTC frequencies, the variance explained increases to 50.9%, with an improvement of 1.4% points (χ^2^(2) = 3.93, *p* = .0001).

The favorable results for social media norms with respect to the baseline hold when introducing non‐linear terms, as modeled by means of restricted cubic splines with three knots (ELP: χ^2^(4) = 4.37, *p* = .0001; BLP: χ^2^(4) = 7.69, *p* = .0001). In ELP, the inclusion of the non‐linear component accounts for a further *R* ^2^ improvement of 0.2%. In BLP, the inclusion of the non‐linear component accounts for a further *R* ^2^ improvement of 0.4%.

In both datasets, we observe that the difference between user counts and raw frequencies for social media corpora are either null or very little (indeed, the Spearman correlation between the raw frequencies and user counts was very high: *ρ* > 0.99). In following discussions, we will thus consider raw frequency counts only. On the other hand, for subtitle‐based frequencies, the contextual diversity metric was clearly better than the raw frequency metric; for subtitle norms, we will refer to the contextual diversity (CD) in our discussion. The superiority of the dispersion measure for subtitle norms, as opposed to social media norms, may depend on the larger units of analyses considered in the former case: subtitles for a given TV program provides a larger document than the sample of tweets collected from a unique user, leading to more opportunities to reuse idiosyncratic vocabulary.

### 4.2 Effect of English variants

**Figure 1:**
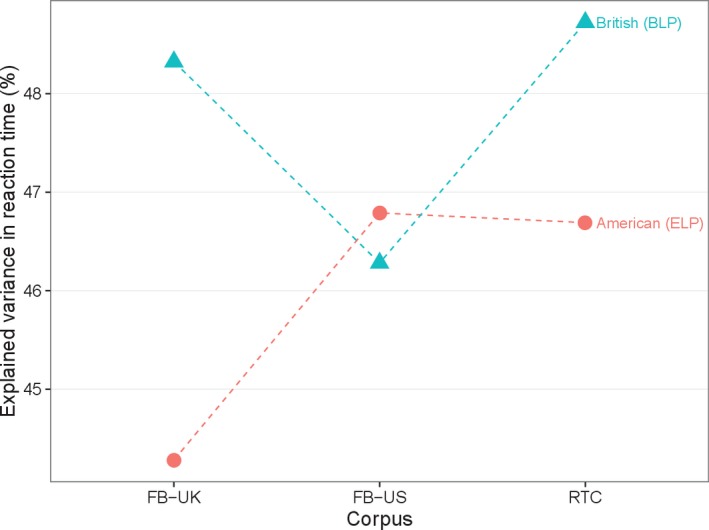
Variance explained by social media frequency norms in the BLP and ELP item sets for American and British English.

Social media frequencies are robust with respect to the particular English variant. Van Heuven et al. (2014) showed that US‐based subtitle frequencies explained the ELP (American English) RTs better than UK‐based subtitle frequencies, and that UK‐based subtitle frequencies explained the BLP (British English) RTs better than US‐based subtitle frequencies. In Fig. 1 we observe that the effect holds for FB‐based frequency values. Indeed, FB‐US has a better performance in the ELP set as opposed to FB‐UK (*z* = 3, *p* = .0027), whereas FB‐UK has a better performance in the BLP set as opposed to FB‐US (*z* = 2.8, *p* = .0051).

The results also suggest that RTC, whose size is comparable to that of FB‐US and FB‐UK, is robust to different variants. Indeed, RTC performance is comparable to that of FB‐US in the ELP set (*z* = 0.17, *p* = .8651), and comparable to that of FB‐UK in the BLP set (*z* = 0.57, *p* = .5687). The same robustness holds when considering a combined FB corpus containing both FB‐UK and FB‐US.

### 4.3 Effect of corpus size

Our initial analyses showed that both FB‐(UK/US) and RTC norms are better predictors of human language processing than previously proposed frequency norms. However, the corpora we used are substantially larger than the previously reported corpora, suggesting that their performance may be due to the increased corpus size. In order to test this hypothesis, we down‐sampled the FB and RTC corpora. Down‐sampling for FB variants was performed at a post‐level; that is, we treated each post as an independent document and sampled full documents. We did not have access to tweet‐level data for RTC; hence, we simulated down‐sampling by treating the frequency of each token as a binomial random variable and taking repeated draws for a desired corpus size (i.e., we sampled at the word‐level). We obtained samples corresponding to 1/2, 1/10, 1/20, 1/100, and 1/200 of the corpus size of both the US‐ and the UK‐variant of the FB corpora and RTC (roughly 500‐, 100‐, 50‐, 10‐, and 5‐million token samples).

**Figure 2:**
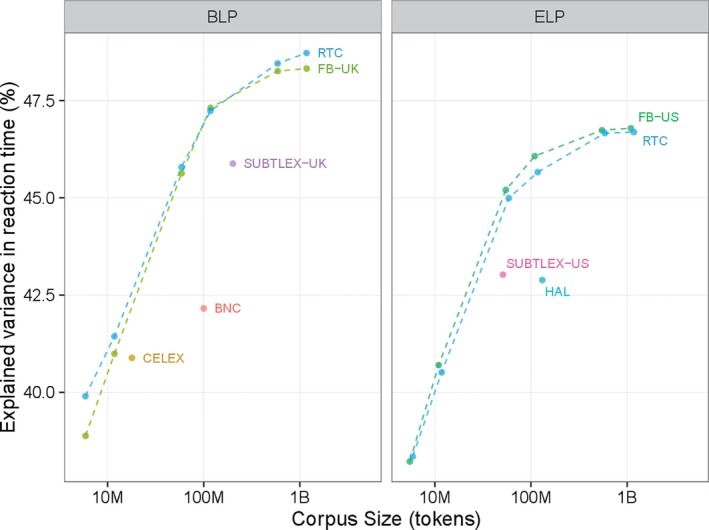
Effect of corpus size on explained variance in reaction times.

The left‐hand side of Fig. 2 represents the variance explained by RTC and FB‐UK for different sample sizes, along with SUBTLEX‐UK,^1^ BNC, and CELEX. First, even at slightly lower sizes, RTC and FB‐UK continue to outperform other frequency measures (in all cases *z* > 2.03, *p* < .0424), with the only exception of CELEX that reach the same performance of the slightly smaller social media subsamples (FB‐UK: *z* = 0.84, *p* = .4009; RTC: *z* = 1.34, *p* = .1802). Secondly, up to roughly 100 million tokens, both RTC's and FB‐UK's performances continue to improve with larger corpus size (FB‐UK: *z* = 2.21, *p* = .0271; RTC: *z* = 1.84, *p* = .0658). Beyond that size, we do not observe any visible difference.

A similar pattern is visible for ELP as well, as shown in the right panel of Fig. 2. Here, we provide the variance explained by RTC and FB‐US for different sample sizes, along with SUBTLEX‐US and HAL. At comparable corpus sizes, RTC and FB‐US outperform other frequency measures (in all cases *z* > 3.4, *p* < .0007). Both RTC and FB‐US‐based frequencies benefit from larger corpus as far as a sample of roughly 50 million tokens is considered (FB‐US: *z* = 5.44, *p* = .0001; RTC: *z* = 5.58, *p* = .0001). After that, increasing the corpus size to 1 billion tokens has diminishing effects.

In their pioneering work on subtitle‐based frequencies, Brysbaert and New (2009, p. 980) claimed that

[A corpus] of 16–30 million words suffices for reliable word frequency norms. In particular, there is no evidence that a corpus of 3 billion words is much better than a corpus of 30 million words. For these sizes, it becomes more important to know where the words of the corpus came from.

Our results corroborate this conclusion as the higher predictive power of the social media norms is not entirely due to the increased corpus size. However, the threshold seems higher for social media corpora which show a performance improvement up to a corpus size of 50–100 million. In line with what we observed for the dispersion measures, the advantage of social media may be due to their small document size that guarantees broader lexical samples and less problem of individuals repeating their idiosyncratic word stock.

### 4.4 Prediction in slow‐response versus fast‐response words

In the previous analyses, we have shown that frequency norms obtained from social media outperformed those based on both traditional corpora and subtitle corpora in predicting reaction times. Their better performance does not depend on the difference in corpus sizes. In the present and the following section, we investigate why this happens, by assessing which items hold the highest difference in terms of performance and how the corpora differ in qualitative terms.

We grouped the words in ELP into 10 deciles based on their RTs, such that the first decile consists of the fastest‐response 10% words (i.e., those with shortest RTs) and the tenth decile consists of the slowest‐response 10% words. For each corpus, we fit a log‐linear model with only the logarithm of the word frequency to predict RTs, and we compute the absolute residuals between the predictions and the actual RT values (let *r*_*C*_(*w*) denote the residual for word *w* when we use corpus *C* for prediction). To compare two corpora, we compute the mean absolute error (residual), separately for each decile: $f_{d}\left(C_{1},C_{2}\right)=\frac{\Sigma_{w\in d}\left(\right|r_{C_{1}}\left(w\right)|-|r_{C_{2}}\left(w\right)\left|\right)}{\Sigma_{w\in d}1}$ where *d* is a decile. If *f*_*d*_(*C*_1_, *C*_2_) is positive, we can say that, on the average, the predictions based on *C*_2_ are better in terms of absolute residuals compared to those of *C*_1_.

**Figure 3:**
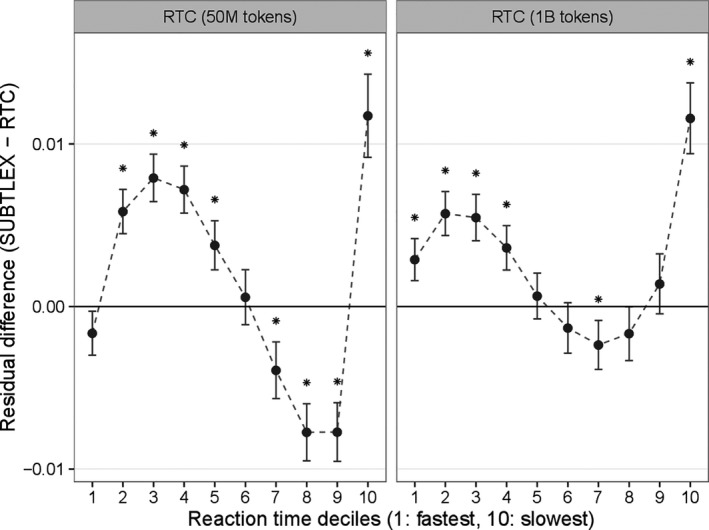
Mean absolute residual differences between RTC and SUBTLEX‐US in reaction time modeling analysis, conditioned by reaction time deciles. Deciles for which a paired *t* test is significant (*p* < .001, using a Bonferroni correction) are marked by an asterisk.

In Fig. 3, we plot *f*_d_ (SUBTLEX‐US, RTC) values for two different sample sizes for RTC, 50 million tokens and 1 billion tokens. Similar‐sized RTC corpus (left‐hand side panel, 50 million tokens) has better predictions for low and very high latency items compared to SUBTLEX‐US. This is despite the fact that there are more unobserved words in RTC (1073), compared to SUBTLEX‐US (572). For mid‐range words, SUBTLEX‐US provides better predictions.

The difference in very slow‐latency words increases when we employ a larger RTC corpus (1 billion tokens), and the SUBTLEX‐US advantage for mid‐range words is much less evident. The results are robust: We find consistent results when running the same comparison between FB‐US and SUBTLEX‐US, as well as when considering the BLP dataset.

### 4.5 Language registers

To obtain a first understanding as to why social media provides better estimates for lexical decision latency, we compare the word frequencies in FB, RTC, and SUBTLEX corpora, and analyze the residuals in RT prediction tasks. Since a thorough content analysis of the social media text is beyond the scope of this study, our aim is not to carry out detailed comparative analysis of the corpora, but rather provide some insights about their differences.

In Table 5, we provide the most over‐represented words in each corpus. For FB‐US and RTC, the comparison is made against SUBTLEX‐US. For SUBTLEX‐US, we provide the comparison against RTC, but the results were very similar when SUBTLEX‐US was pitted against FB‐US. To give a representative example set, we show the top‐10 words for each corpus, taken from different percentile intervals of reaction times. We used log‐likelihood ratio statistic (Dunning, 1993) to extract the words whose distributions across the two corpora deviate the most from the null model where the expected frequencies are the same.

**Table 5:** Over‐represented words in each corpora, according to log‐likelihood score (*G* ^2^), for different reaction time quintiles

Quintile	FB‐US (w.r.t. SUBTLEX‐US)	RTC (w.r.t. SUBTLEX‐US)	SUBTLEX‐US (w.r.t. RTC)
1 (fastest 20%)	my, day, year, for, today, love, friends, its, so, being	my, its, love, follow, today, day, new, though, for, so	you, sir, here, right, it, know, he, okay, that, do
2	and, real, but, wit, part, awesome, miss, ass, work, tonight	real, wit, at, but, awesome, ass, watching, miss, and, weekend	mean, won, were, got, father, minute, yeah, doing, honey, come
3	gonna, birthday, gotta, thankful, bout, prayers, status, season, evening, wish	gonna, via, gotta, followers, bout, wish, birthday, posted, season, boo	him, little, three, supposed, ought, hundred, yours, mister, colonel, enough
4	am, haha, mommy, hubby, momma, disclose, contained, posting, ma, goodnight	haha, ma, wow, huh, awkward, rite, ad, goodnight, congrats, mum	something, ahead, pardon, discuss, sweetheart, downstairs, warrant, champagne, brilliant, assure
5 (slowest 20%)	a, thru, their, ugh, yea, ah, awhile, tech, hating, auntie	a, twitter, yea, ah, ugh, thru, follower, subscribed, tech, awhile	haven, sergeant, ashore, scare, adjourned, missiles, vanquish, slipstream, potion, hostage

We observe that the over‐represented words in SUBTLEX include terms of address such as *you, it, he, sir, honey, sweetheart, mister* and/or words that can be used in conversational context such as *yeah, pardon, okay*. Moreover, the words *sergeant, colonel, missiles, hostage*, and *vanquish* reveal the fictional nature of the sources used for SUBTLEX, such as movies and TV shows.

On the other hand, the words over‐represented in FB‐US and RTC are more related to social ties in informal context (*mommy, hubby, momma, ma, auntie, mum*), feelings (*love, miss, thankful*), personal matters or words about the now or immediate future (*birthday, weekend, work, today, tonight*).

In order to obtain more clear evidence concerning register differences between the corpora, we group individual words according to their LIWC categories and subcategories and compare the aggregated corresponding frequencies. LIWC (Linguistic Inquiry and Word Count) is a widely used text‐analysis tool/dataset that provides sets of words under different psycholinguistically relevant categories (Pennebaker, Francis, & Booth, 2001). In terms of the number of word types encountered in ELP, the most common categories and example words are given below.

1. Affective processes (including subcategories of positive and negative emotions): happy, cried, abandon
2. Relativity (including subcategories motion, space, and time): area, arrive, down, car, in, season
3. Biological processes (including subcategories body, health, sexual, and ingestion): eat, blood, cheek, clinic, love, pizza

**Figure 4:**
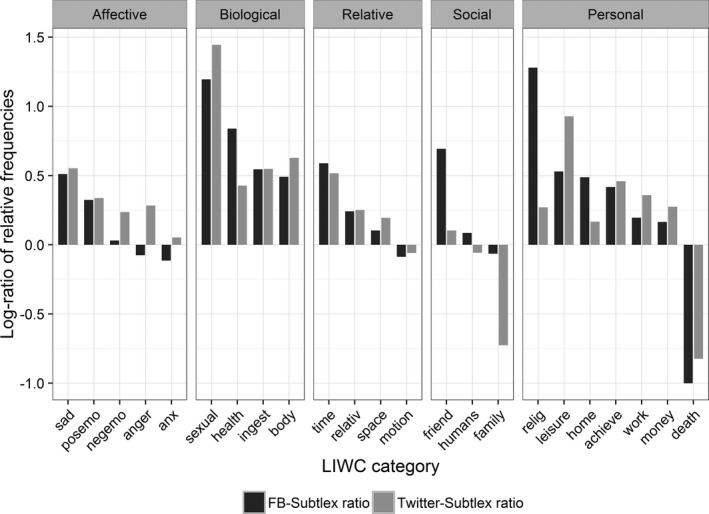
Log frequency ratio of SUBTLEX‐US and social media frequencies. The dark bars are log_2_(FB‐US/SUBTLEX‐US), and the light bars are log_2_(RTC/SUBTLEX‐US).

In Fig. 4, we provide the log odds ratios of the LIWC subcategories that fall under one of the above three main categories, plus two categories that we deem to be particularly interesting for the present comparison: social (e.g., *mate, daughter, friend, baby*) and personal (e.g., *job, earn, cook, church*). Only subcategories with more than 30 words observed in ELP dataset are considered. Further information concerning within‐subcategories variance can be found in the Appendix (Table A2).

First, we observe that the differences in the two social media corpora with respect to SUBTLEX‐US are strikingly similar, both across the subcategories and high‐level categories or LIWC. Second, we observe substantial across‐categories differences between SUBTLEX and social media. Words in biological processes, which are mostly person/body oriented, are more represented in FB‐US and RTC. Also, words in the “personal” category are over‐represented in social media, with the exception of *death*—which is understandable given that death is not such an uncommon event in movies and, to a certain degree, may be a taboo topic in the social media we considered.

The result of the present section indicates that the social media and subtitle‐based corpora indeed capture different linguistic registers. Along with the better performance of social media norms observed in the previous sections, these data suggest that the linguistic register captured by social media may be more representative of how words are stored in the cognitive system. In other terms, the way language is used in social media parallels more closely with the way language is represented and processed in the human mind (as opposed to subtitles or traditional corpora). As a result, social media data are more apt at defining how salient a linguistic input is at the mental level.

## 5 Discussion

Obtaining word frequency norms that better explain language processing data is an ongoing effort in the psycholinguistics community (van Heuven et al., 2014). In this study, we introduced two social media‐based word frequency norms (based on Facebook and Twitter) and showed how they obtain the new state‐of‐the‐art performance in predicting reaction times in lexical decision tasks.

The results in favor of social media data are robust. They are observed for two independent frequency norms based on two different social media sites. The social media frequency norms can increase the variance explained in reaction times by more than 3%, even against a very strong baseline model including word‐form properties as well as formerly proposed frequency norms. These are substantial improvements, considering that many of the variables studied in psycholinguistics explain 1% of the variance after controlling for word similarities and word‐form properties (Brysbaert et al., 2011; van Heuven et al., 2014).

Crucially, the improvement is not simply due to the increased size of the corpora. Even when considering smaller‐size subcorpora, Facebook and Twitter norms continue to provide better estimates in comparison to other databases. Over and above the higher explained variance in reaction times, our analyses highlighted properties of social media data that are interesting for psycholinguistic purposes. These properties concern the robustness to language variants, the potential to capture aspects of less familiar words, and the particular language register used.

Social media norms provide a mix of different English variants. Both RTC and FB have good results across the tests for both American and British variants. For this reason, social media may constitute the ideal choice of frequency norms for experimental situations in which the considered language variant is mixed, unclear, or does not have available norms.

We also found that the good results of social media norms are mostly due to items that elicit very long or very short reaction times. Long‐response words are particularly interesting. These rare, unfamiliar words are difficult to capture through corpus statistics. On one hand, they manifest a certain degree of variance in the associated behavioral responses. On the other hand, this variance is rarely paralleled in corpora, where rare terms may be found only once (*hapax legomena*), if at all (Church, 2000). This drawback is less evident in social media norms. A possible explanation for this phenomenon may be found in the effect of *word prevalence*. This measure, defined as the count of people knowing a given word, has been shown to be an ideal predictor of slow‐response words (Keuleers, Stevens, Mandera, & Brysbaert, 2015). Frequencies based on social media may be more strongly related to word prevalence than those based on traditional corpora. Whereas the latter focused on documents produced by a limited number of expert authors, the former collects language examples from an extremely large sample of speakers. As a consequence, in social media data higher frequency would also indicate that the considered word is known by many people: The association between frequency and prevalence (and the consequent good performance for slow‐response items) would depend on the very nature of the proposed frequency norms. This intuition is indeed supported by the extremely high correlation between raw frequency and user counts in Facebook and Twitter data.

Additionally, we observed that the social media and subtitle corpora may be characterized by different language registers. SUBTLEX contains more conversational words (interjections), whereas social media contain more words related to personal matters (biological, personal, feelings). This is surprising. Before starting the analyses, our expectations were that social media would have provided good examples of natural language exchanges, thus over‐representing the “conversational style” also captured by subtitles. Contrary to our expectations, we found that (a) social media data capture a register focused on the personal sphere, and (b) conversational aspects are over‐represented in subtitle corpora. If the former result (a), in retrospect, makes sense (we collected data on Facebook status updates, and excluded responses to the status updates), the latter (b) goes against the assumption that lexical representations should be modeled on conversational data, which are in turn supposed to be closer to the natural language experience (Brysbaert & New, 2009).

Why should a language register that focuses on the personal sphere provide better predictors of language processing? A possible answer is offered by the results on the processing of self‐referential and non‐self‐referential words (Herbert, Herbert, Ethofer, & Pauli, 2011; Blume & Herbert, 2014). These findings indicate that the potential self‐referentiality is rapidly evaluated in language processing, and that self‐referential words are particularly salient when considering both brain and behavioral responses. This aspect, rather overlooked in the psycholinguistic research on visual word recognition, may explain why a register focused on the personal sphere is predictive of language processing and should be more thoroughly considered in future research.

Certainly, the present evidence does not imply that the conversational register is not important at all when collecting lexical frequencies. The good performance of subtitle corpora in predicting lexical decision reaction times clearly indicates that conversational aspects play a crucial role in language processing. Indeed, given that social media and subtitles capture very different linguistic domains, they may be seen as complementary resources in the enterprise of creating good frequency norms for psycholinguistic purposes. A preliminary analysis seems to confirm this intuition: When considering frequency values obtained by averaging subtitles and Facebook norms, a further improvement can be observed in the prediction of response latencies (1.09% for American data, 1.93% for British data). Further investigation in this respect is certainly needed—it is probably the case that the two norms do not provide equal contribution to the performance improvement, and hence a weighted average of the two norms (with weights estimated in a principled way) could be the best option. We leave this question to future research. However, this first piece of evidence suggests that, for methodological purposes, a combination of the two measures may be the ideal solution.

In conclusion, the present paper examines a new source for extracting word frequencies for psycholinguistic experiments, namely social media like Facebook and Twitter. These resources have both quantitative and qualitative advantages in comparison to previously described methods. On one hand, they constitute extremely large and always increasing sources of linguistic data for a large number of different languages. On the other hand, they provide examples of natural, contemporary, and spontaneous linguistic productions for a wide range of topics, as opposed to the limited, scripted, and edited nature of existing databases. Empirical results support the reliability of social media norms in lexical decision studies. We therefore encourage the usage of these norms in psycholinguistic experiments. To this purpose, we release datasets for both Facebook and Twitter data, that can be downloaded from http://www.marcomarelli.net/resources and ideally complement other recent resources based on social media (e.g., the frequency norms by Gimenes and New (in press), based on Twitter). Furthermore, the impact of the present paper is not limited to the methodological side. The results we observed concerning the register used in social media were surprising, and question traditional assumptions as to which aspects are to be considered when working in psycholinguistics. We hope that this first exploration in social media lexical frequencies will encourage the usage of these resources in psycholinguistics and motivate future studies in the field.

## Note

1 In this analysis we are interested in comparing the effect of corpus size. Since contextual diversity, as number of TV programs, is not directly affected by corpus size, we used raw frequency metrics for SUBTLEX as well as all other corpora.

## Appendix 1

### 1.1

**Table A1:** Absolute correlation and variance explained of various measures with respect to the lexical decision latencies from the entire English Lexicon Project

Corpus	Pearson Correlation	*R* ^2^
HAL	0.611	0.392
SUBTLEX‐US	0.648	0.447
SUBTLEX‐US CD	0.654	0.454
RTC	0.684	0.490
FB‐US	0.676	0.480
Baseline		0.623
Baseline + RTC		0.632
Baseline + FB‐US		0.632
Baseline + FB‐US + RTC		0.633

Baseline refers to the model that includes HAL, SUBTLEX‐US, SUBTLEX‐US CD, number of syllables, and number of letters in the word. Frequency values and response latencies are log‐transformed.

**Table A2:** Standard error of the mean (*SEM*) for log ratio relative frequencies across words within a LIWC subcategory. *SEM*s are reported for both log_2_(FB‐US/SUBTLEX‐US) (*SEM* FB) and log_2_(RTC/SUBTLEX‐US) (*SEM* RTC)

Category	Subcategory	Word Count	*SEM* FB	*SEM* RTC
Social	Family	52	0.266	0.193
Social	Friend	39	0.261	0.215
Social	Humans	44	0.259	0.222
Affective	Posemo	532	0.070	0.062
Affective	Negemo	738	0.050	0.050
Affective	Anx	135	0.128	0.123
Affective	Anger	254	0.082	0.084
Affective	Sad	168	0.096	0.108
Biological	Body	324	0.083	0.085
Biological	Health	263	0.095	0.094
Biological	Sexual	100	0.157	0.152
Biological	Ingest	201	0.094	0.084
Relative	Relativ	963	0.043	0.043
Relative	Motion	305	0.073	0.080
Relative	Space	372	0.069	0.066
Relative	Time	273	0.085	0.081
Personal	Work	385	0.084	0.083
Personal	Achieve	265	0.085	0.084
Personal	Leisure	354	0.083	0.081
Personal	Home	180	0.135	0.116
Personal	Money	228	0.099	0.094
Personal	Relig	141	0.177	0.148
Personal	Death	88	0.147	0.149
